# Structure and Ultrastructure of Three Oak Leaf Galls: *Cynips quercusfolii* L., *Neuroterus numismalis* Geoffroy and *Cynips longiventris* Hartig

**DOI:** 10.3390/insects16020173

**Published:** 2025-02-06

**Authors:** Leszek S. Jankiewicz, Marzenna Guzicka, Agnieszka Marasek-Ciołakowska

**Affiliations:** 1 The National Institute of Horticultural Research, Konstytucji 3 Maja 1/3, 96-100 Skierniewice, Poland; leszekjankiewicz555@gmail.com; 2 Institute of Dendrology, Polish Academy of Sciences, Parkowa 5, 62-035 Kórnik, Poland

**Keywords:** Cynipidae, Hymenoptera, plant gall anatomy, plant gall ultrastructure, *Quercus robur* L.

## Abstract

The structural and ultrastructural characteristics of galls induced by three species of insects parasitizing on oak leaves (*Quercus robur* L.) were examined utilizing light and fluorescent microscopes, as well as scanning and transmission electron microscopes. The tissues of the investigated galls exhibited marked differences from those of a typical oak leaf. In the *Cynips quercusfolii* L. gall, the larval chamber in its final stage was formed from the remnants of dead cells that remained after larval feeding on the gall nutritive tissue. The cells of the gall nutritive tissue and the cells of the gall parenchyma exhibited diametrical differences: the former contained dense cytoplasm and had large nuclei and nucleoli, whereas the latter displayed sparse cytoplasm, prominent vacuoles, and very small nuclei. The region of coalescence between the gall stalk and leaf tissues has been described. In *Neuroterus numismalis* Geoffroy gall, the early developmental phases have been described in detail. The external gall tissues resembled periderm, whereas periderm does not normally occur in leaves. In the cytoplasm of *Cynips longiventris* Hartig gall, different bodies were found, including organized smooth endoplasmic reticulum; however, not all of the observed structures were definitively classified.

## Simple Summary

Galls are plant growth anomalies caused by bacterial, viral, fungal, and animal parasites, although the most complex structures are formed by insects. Their induction, development, and gall–host plant interactions remain insufficiently understood at the structural level. We posit that the enhanced comprehension of gall structure and ultrastructure will facilitate our understanding of the galling process. The anatomy and ultrastructure of galls induced by the three species of cynipid wasps on oak leaves (*Quercus robur* L.) were compared with those of normal oak leaves. The area of coalescence between leaf tissues and gall tissue was examined, as well as the walls of the larval chamber during pupation time. The galls of each species exhibited specific anatomical and ultrastructural characteristics. We found that the type of storage material in nutritive tissue could differ between the galls of each wasp species, as well as between the galls of the same species in different stages of development. A notable finding was the similarity of *N. numismalis* gall external tissues to periderm tissue that typically does not occur on leaves. This suggests that galling insects can induce the formation of certain host tissues in atypical locations.

## 1. Introduction

Various groups of organisms have the ability to induce plant galls. Notable examples include crown galls triggered by *Agrobacterium tumefaciens* [1] and root galls caused by parasitic nematodes [2]. From the perspective of plant morphogenesis, the most intriguing galls are those induced by insects of the family Cynipidae because of their complexity, variability of forms, and the large number of species involved [3,4]. The process of gall formation is like an experiment conducted by nature on plant morphogenesis. The longevity of this phenomenon is evidenced by the discovery of gall-like structures in plant fossils dating back to the late Paleozoic and early Mesozoic eras [5,6]. Consequently, the evolution of plant–arthropod interactions, resulting in gall formation, has persisted long enough to produce the complex structures observed in the present day. The induction of cynipid gall is difficult to explain [6,7,8]. The insect provides the stimulus for gall formation only to one or a few cells of the host organ, and these cells multiply rapidly, producing cells that redifferentiate to form a gall, an organ-like entity that provides shelter and food for the developing larva [6,8,9,10]. The gall of each species of insect of the Cynipid family has a specific, original form and structure [3,4,11]. The cynipid galls presented here are composed of various tissues and are as complex as any plant organ. Additionally, the physiology of gall tissues, biochemical and metabolic changes, and protein expression differs noticeably from that of normal host tissues [7,9,12,13,14].

Among the hypotheses trying to explain the phenomenon of gall formation [7,15], the most favored one states that the hatched larva or even the egg [16] introduces the hormone-like stimuli to the host plant, and these substances induce gall formation [17,18,19,20]. One of the works that supports this hypothesis is that of Bartlett and Connor [21]. They injected different hormones into the pepper (*Capsicum annuum*) leaf petioles. A mixture of cytokinin and auxin was most effective in stimulating gall-like tissue swelling. Recent studies have shown that both auxin [22] and cytokinins [23] are abundant and widespread among insects, both in gall inducers and non-inducers. It has also been proposed that the fluid secreted by females into plant tissue during oviposition plays a crucial role in gall initiation [24].

The mediation of some microorganisms in the transfer of the genetic material from an insect to the host leaf was also taken into consideration [25]; however, it was proved experimentally [26]. One of the serious difficulties in the investigations of the galling process is the scarcity of saliva produced by most of the galling insects [27].

The species of Cynipid gall wasp (Hymenoptera, Cynipidae) have two generations: both agamic and sexual generations (heterogony). These two generations can differ in the morphological structure of the adults, the plant organ attacked, and the structure of the galls formed [7]. Galls in which the dioecious generation develops occur in spring and/or early summer, whereas galls in which the monoecious generation develops occur in late summer and fall [28].

The present study focuses on the anatomical and ultrastructural properties of galls induced by the asexual generation of three species of cynipid wasps: *Cynips quercusfolii* L., *C. longiventris* Harting, and *Neuroterus numismalis* Geoffroy. They induce the gall on the oak *Quercus robur* L. (Fagaceae), and they are highly specific to this taxon; they are monophagous and associated with the leaves of the host. The galls of these inducers sometimes occur simultaneously on the same leaf, but they always preserve the morphotype. We have compared the gall structure to that of a typical oak leaf. The aim of the present study was to broaden our knowledge on the unique characteristics of each gall, especially in terms of the connection between structure and function and larval needs. Our idea was that without the detailed description of the structure and ultrastructure of the gall, it would be difficult to understand gall induction and development.

## 2. Materials and Methods

### 2.1. Plant Material

**Figure 1:**
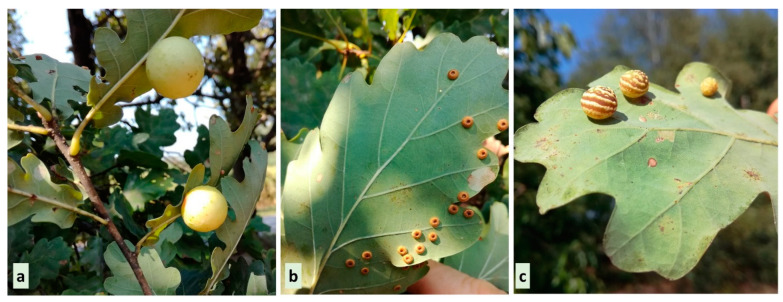
Three oak galls induced by the agamic generation of (**a**) *Cynips quercusfoli* L., (**b**) *Neuroterus numismalis* Geoffroy, and (**c**) *C. longiventris* Hartig. Picture taken in mid-August.

The plant galls, *Cynips quercusfolii* L., *Neuroterus numismalis* Geoffrey, and *C. longiventris* Hartig (Hymenoptera, Cynipidae), described in this paper are some of the most common in Poland (Figure 1) [29]. They were collected in an oak–pine forest aged about 5–25 years from Bolimów Landscape Park near Skierniewice, Poland (2°0′32.4″ N, 20°11′49.2″ E). The oak leaves taken for analysis were healthy and fully developed, with or without galls. The galls induced by the same-sex generation of cynipid wasps were sampled at different stages of their development from several trees (July 19th, August 15th, September 18th). The diameter of the galls induced by *C. quercusfolii* ranged from 1.3 to 2.6 cm; the diameter of the *N. numismalis* gall was approximately 1.2–3 mm, while the diameter of the *C. longiventris* gall was approximately 8–12 mm. The plant material was collected from trees growing in humus-rich soils, either in full sunlight or lightly shaded.

### 2.2. Cytochemical Studies in Light (LM) and Fluorescence (FLM) Microscopes

The plant materials for light microscopy (LM) were prepared according to the methods described by Jankiewicz et al. [30,31] and Pathan et al. [32]. A minimum of 3–4 samples from distinct trees were obtained for each investigation. Briefly, the galls with oak leaves were fixed in a chromic acid–acetic acid–formalin mixture (CrAF), dehydrated in a graded series of ethanol, and embedded in paraffin. Paraffin blocks were sliced using rotary Zeiss microtome into 10 μm thick sections and after staining with safranin and fast green or with Sudan III. Microscope preparations were observed with a Zeiss Axio Imager AI equipped with DIC. In this method, the chromosomes, nuclei, and lignified walls appeared to be red (due to the effect of safranin), whereas the cytoplasm and cellulosic cell walls appeared to be green (effect of fast green).

For nucleus detection, the DAPI dye (4′,6-diamidino-2-phenylindole) and staining protocol of Ruzin [33] were utilized. Hand-cut slices of fresh oak gall material were prepared for staining. The Leica SP5 fluorescent microscope was used to observe the slides under UV (365 nm) excitation, which results in blue fluorescence when bound to chromatin. Lignified cell walls might also be discernible as lignin exhibits blue autofluorescence under UV excitation.

### 2.3. Scanning Electron Microscope (SEM)

The typical leaves, with or without galls, were fixed with a CrAF mixture (comprising chromic acid, acetic acid, and formalin). The samples were then dehydrated in ethanol, desiccated using critical point drying with CO_2_, and sputter-coated with gold [32]. The micromorphology of the gall surface, the oak leaf surface, and the internal structure of the galls were analyzed using the JEOL JSM 6390LV scanning electron microscope, at the Mossakowski Medical Research Centre, Polish Academy of Sciences in Warsaw.

### 2.4. Fixation, Dehydration, Embedding, Cutting, and Contrasting Ultrathin Slices for Observations in Transmission Electron Microscope (TEM)

Fragments of plant material (2 × 2 mm) were fixed in 2.5% glutaraldehyde for 12 h at 4 °C in 0.1M cacodylate buffer (pH 7.4) [34]. They were then rinsed several times in the same buffer for 12 h. Subsequently, the material was treated with 1% aqueous solution of osmium tetroxide for 4 h. After dehydration in ethanol and propylene oxide [35], the material was embedded in an epoxide resin mixture (Spurr) [36]. It was then cut into semi-thin sections of 2 µm and ultrathin sections (approximately 80 nm) using the LKB ultramicrotome. The ultrathin sections were then contrasted with uranyl acetate and lead citrate according to Reynolds [37]. The studies were conducted using the JEOL JEM-1200EX transmission electron microscope.

## 3. Results

### 3.1. The Ultrastructure of an Oak Leaf

**Figure 2:**
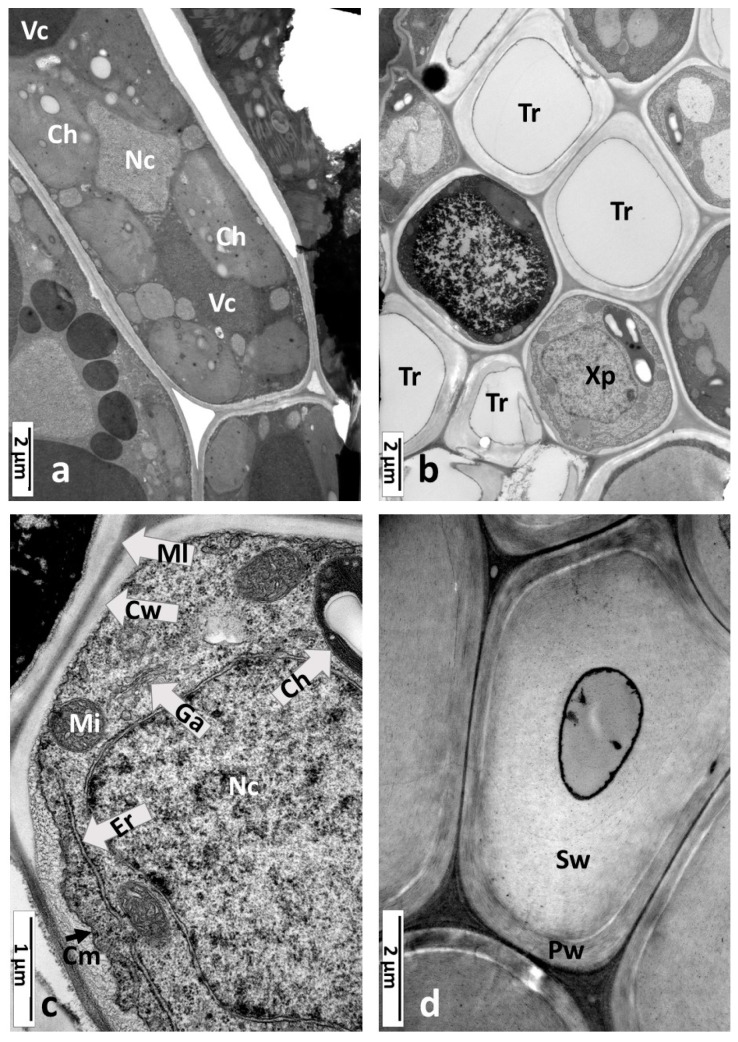
Typical oak leaf cells (TEM). (**a**) The cells of palisade parenchyma: Ch, chloroplasts with starch grains (white); Nc, nucleus; Vc, vacuoles—sometimes they contain electron-dense material (probably tannins). (**b**) Small vein of the leaf blade in a cross-section; Tr, tracheary elements; Xp, xylem parenchyma cell. (**c**) Xylem parenchyma cell—detail of Figure 2b; Ch, chloroplast with a starch grain; Cm, cell membrane (plasmalemma); Cw, cell wall; Er, endoplasmic reticulum; Ga, Golgi apparatus; Mi, mitochondria; Ml, middle lamella; Nc, nucleus. (**d**) Phloem fibers in a cross-section through a small leaf vein. Pw, primary wall; Sw, secondary wall.

The ultrastructure of a healthy oak leaf without galls was analyzed using TEM (Figure 2) and served as the control. The cells of the palisade parenchyma had mostly oval elongated shapes and were thin-walled (0.26–0.29 µm). Typical cells were approximately 30–40 µm long and 5–7.5 µm wide (Figure 2a). They contained an irregularly shaped nucleus with diffuse chromatin (the nucleoli were not visible in this section). More than one vacuole is often present in cells. Vacuoles frequently contained electron-dense materials (tannins). Large chloroplasts containing small starch grains were localized adjacent to the cell walls and accompanied by oval or spherical mitochondria with cristae. Chloroplasts occupied a large part of the internal space of the palisade cells. The cells of the spongy parenchyma were similar but had an irregular shape and were loosely arranged. In the cross-section of a small vein of a leaf blade (Figure 2b), there are two groups of cells with thick walls, empty inside, so they were classified as tracheary elements. They were accompanied by xylem parenchyma cells. Some cells had electron-dense precipitations in their content or only in the vacuole (probably tannins). It is probable that the cell with a marked deposit of tannins in the middle of Figure 2b is a phloem cell. The xylem parenchyma cell is shown separately in Figure 2c. It had a large nucleus with diffuse chromatin and a nucleolus (not visible in this fragment). There was a chloroplast with large starch grains and osmophilic stroma with some plastoglobuli. Additionally, mitochondria with distinct cristae were observed, along with the endoplasmic reticulum. The phloem fibers accompanied the vascular tissue (Figure 2d). They had a typical structure, with very thick secondary walls. The fiber cells in the analyzed phase of the leaf development had remnants of cytoplasm in their lumens.

### 3.2. The Gall of Cynips quercusfolii

#### 3.2.1. The Area of Coalescence Between Leaf Tissues and Gall Tissue

**Figure 3:**
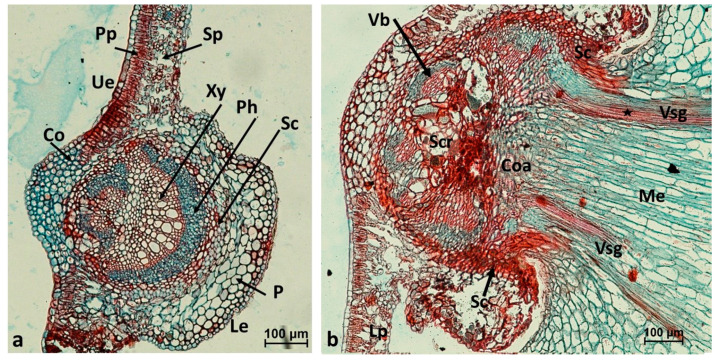
(**a**) Cross-sections of an oak leaf (Quercus robur) cut at the midrib. (LM, fast green + safranin). Pp, palisade parenchyma; Sp, spongy parenchyma; Ue, adaxial epidermis; Co, collenchyma; Xy, xylem; Ph, phloem; Sc, sclerenchyma; P, parenchyma and the collenchyma of the vein; Le, abaxial epidermis. (**b**) The place of coalescence of the *C. quercusfolii* gall tissues with those of oak leaf; Lp, leaf mesophyll; Coa, place where the vascular tissues of the gall (Vsg) meet with those of the leaf; Vb, vascular bundles which supply the distal parts of the leaf; Sc, sclerenchyma caps surrounding the place of coalescence; Scr, a central group of large and small sclerenchyma cells. Me, gall parenchyma in the center of gall stalk; *, spiral wall enforcements.

The gall of *Cynips quercusfolii* usually develops close to a vein. The comparison of the location where the leaf tissues coalesce with those of the gall stalk with the structure of healthy host leaf shows that there are several similar features. For instance, collenchyma is present in both cases in a similar position (Figure 3). The structure of the place of coalescence corresponds to its complex function. The vascular tissue of the stalk connects the leaf vascular tissue with the gall vascular tissue. The vascular bundles can only pass through the coalescence place. Their role is probably to supply the distal parts of the leaf blade (those behind the gall insertion). The central part of the coalescence shows large or small suberized cells of unknown function, possibly mechanical. The Vsg vascular tissues of the gall strands formed a ring around the gall stalk. These vessels showed spiral wall enforcement and were very narrow, similar to the vessels in the primary xylem of the leaf. In the center of the gall stalk, one may see parenchyma with elongated cells, formed from the fundamental meristem. The whole coalescence region was surrounded by the cap of sclerenchyma.

#### 3.2.2. The Gall of *Cynips quercusfolii* L. During Pupation Time

**Figure 4:**
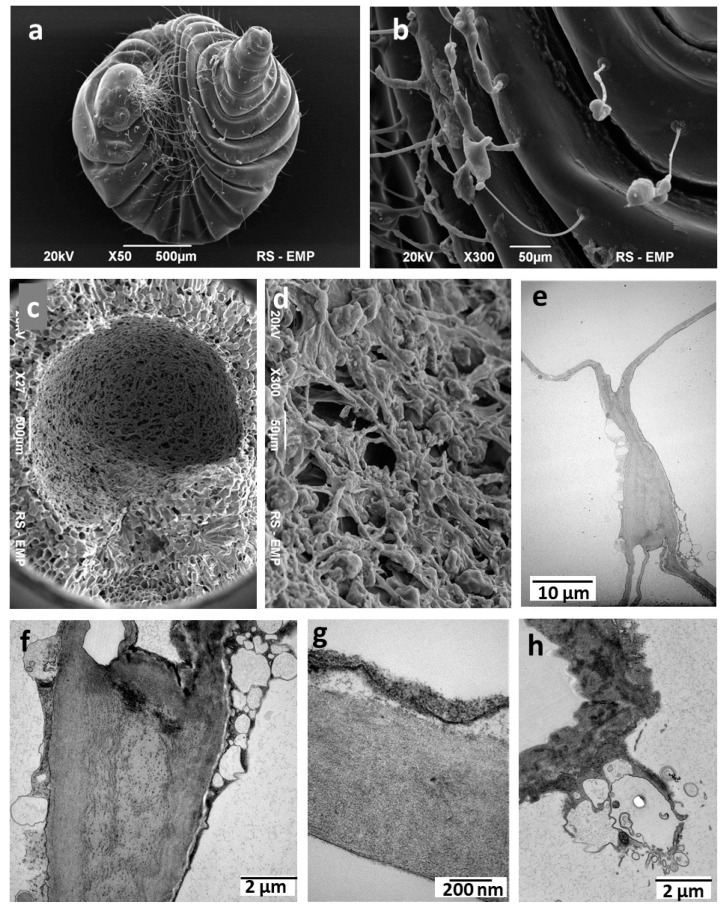
Cynips quercusfolii ((**a**–**d**) SEM, (**e**–**h**) TEM). (**a**) The pupa taken from the larval chamber in September. (**b**) The hairs on the skin of the pupa. (**c**) Larval chamber at a pupation time. (**d**) Net-like structure of larval chamber wall in higher magnification. (**e**) Fragment of the giant cells of a mature gall. (**f**) Fragment of the wall thickening at the junction among four cells. (**g**) Structure of the thin wall of a giant cell. (**h**) In some cells, (rarely) some sectors of the wall had very irregular construction.

The gall when the pupa was already formed is shown in Figure 4a–d. At this stage, the consolidation of cellular debris and deceased cells occurred within the larval chamber (Figure 4c,d). This net was relatively thick and composed of the remains of several layers of nutritive cells that had been consumed successively by the larva. The TEM images in the present study confirmed these observations (Figure 4e,g). However, the thickenings of the cell walls where three or four cells of *C. quercusfolii* gall converged were interesting (Figure 4e,f). Thickening was observed at several locations in the gall tissues. Their role may be to strengthen the parenchyma structure.

#### 3.2.3. Gall Parenchyma and the Nutritive Tissue of *Cynips quercusfolii*

**Figure 5:**
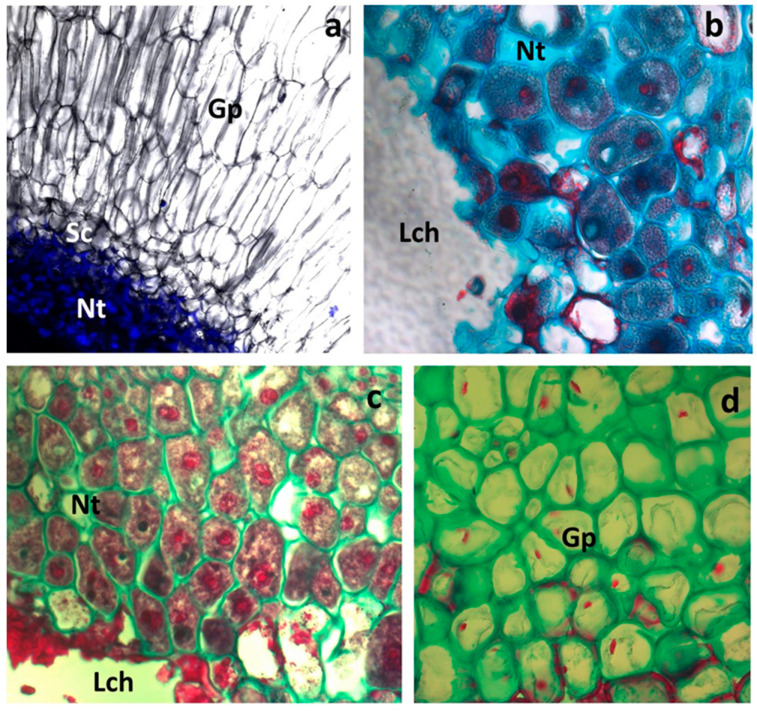
The gall of Cynips quercusfolii. (**a**) The contrast between nutritive tissue (Nt) and the gall parenchyma (Gp) (they are separated by a band of sclerenchyma—Sc), Lch, larval chamber; (FLM, stained with DAPI). (**b**–**d**) Slides stained with fast green and safranin (the cytoplasm, nuclei, and nucleoli are red; the cellulosic walls are green) (LM). (**b**) Nutritive tissue alone at greater magnification. (**c**) Nutritive tissue (Nt) (**d**) Gall parenchyma.

Contrast between the gall parenchyma and nutritive cells was observed using the FLM. The cells of the gall parenchyma (Figure 5a,d) had large vacuoles and scarce parietal cytoplasms. They had very small nuclei; the nucleoli have not been perceived. In contrast, the cells of nutritive tissue (Figure 5b,c) had very dense cytoplasm and contained large nuclei and nucleoli (usually deep red or reddish in this method of staining). The vacuoles were small and, frequently, they were not visible. The cell walls were cellulosic, not lignified, except for the walls of a few sclerenchyma cells (see bottom right corner in Figure 5d). Based on the size of the nuclei, nutritive tissue cells contain a substantial amount of nucleic acids. Moreover, the presence of large nucleoli in these cells (Figure 5b,c) suggests that they were a source of intense protein synthesis.

### 3.3. Gall of Neuroterus numismalis

#### 3.3.1. Young Gall of *Neuroterus numismalis*

**Figure 6:**
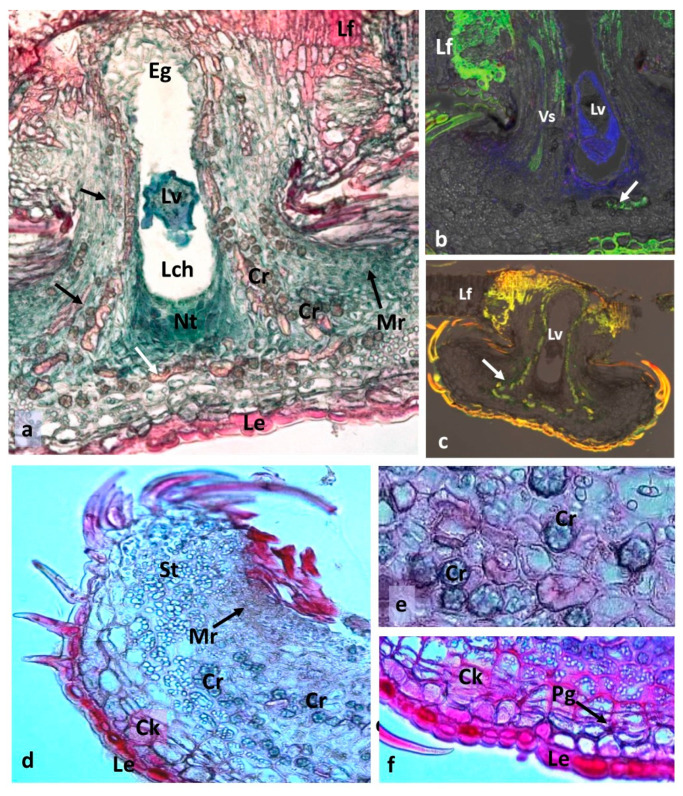
The gall of *Neuroterus numismalis*. (**a**) Early stage of gall development (diameter of the gall about 1.2 mm (collected July 19th)). The larva (Lv) had already left the place where the egg had been laid (Eg) and had moved into the larval chamber (Lch). The abandoned place in the leaf had already been partially cicatrized; Lf, the tissues of the leaf situated around the gall stalk were partly lignified (red color); Cr, crystals, Mr, meristematic region showed by black arrows, so as not to cover the meristematic cells; Le, lower epidermis; Nt, nutritive tissue; white arrow, sclerenchyma capsule starts to form. (LM, polarized light, fast green + safranin). (**b**) Early stage of gall development in FLM. Green fluorescence, lignin; blue, protein; white arrow, beginning of the formation of sclerenchymatous capsule, Vs, vascular strands (**c**) Some early lignified elements in a gall (yellow-brown due to safranin): lower and lateral epidermis, leaf tissues near the gall stalk and sclerenchymatous capsule (white arrow). (**d**) Marginal part of a gall rich in starch (St). (**e**) The shape of the crystals. (**f**) The cork (Ck) starts to be formed; Pg, phellogen (showed by black arrow).

In the early stage of *N. numismalis* gall development (July 19th; diameter of the gall approximately 1.2 mm), soon after the larva had hatched and started moving into the newly formed tissues of the gall, a conspicuous amount of nutritive tissue with very dense cytoplasm was observed (Figure 6a). The oviposition site started to be cicatrized (Eg). The location of the meristematic region near the gall stalk was surprising; we expected it to be located at the margin of the gall disk. The meristematic region was gradually displaced toward the margins of the gall disk during gall development (compare Figure 6a,d). Heavy sclerification of the leaf tissues around the gall stalk was observed (Figure 6a, reddish color). This may contribute to the strong anchorage of the gall stalk in leaf tissues and prevent precocious and undesirable detachment. Strong green luminescence (indicating lignification) of the leaf tissues in Figure 6b confirms this statement. The nutritive tissue near the larva shows blue luminescence, indicating the accumulation of proteins. In the gall presented in Figure 6c, the lignified tissues showed brown-yellow luminescence (due to added safranin). In addition to the prominent lignification of leaf tissue near the gall stalk, the gall epidermis and the hairs showed distinct lignification. The sclerenchymatous capsule that enclosed the larval chamber starts to be visible (white arrow). In the marginal part of the gall, there were large deposits of starch in this part of the gall (Figure 6d). The meristematic region is already close to the margin of the gall. Figure 6e shows the shape of crystals of calcium oxalate (CaOx), which accompany the gall sclerenchyma. In Figure 6f, the initial stage of cork layer formation is visible.

#### 3.3.2. Growing Gall of *Neuroterus numismalis*

**Figure 7:**
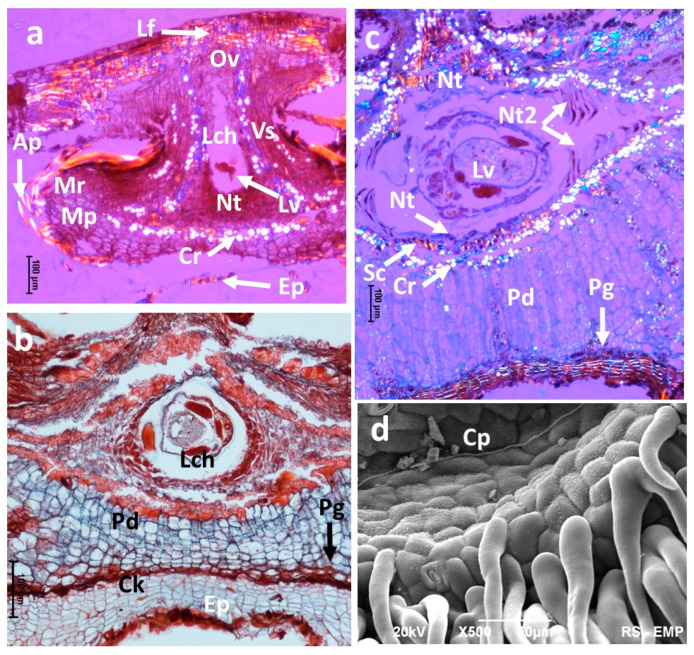
Young *Neuroterus numismalis* galls on oak leaf. (**a**,**c**) LM, polarized light, Sudan III; (**d**) SEM. (**a**) Very young gall (July 19th), multiple calcium oxalate crystals accompanying the gall sclerenchyma that surrounded the larval chamber: Ap, appendices (hairs); Mp, marginal parenchyma; Mr, meristematic region; Ov, partly cicatrized place where egg was laid. Lf, leaf tissue; Lch, larval chamber; Vs, vascular strands; Lv, larva; Nt, nutritive tissue; Cr, crystals; Ep, epidermis (**b**) Gall older by 10 days: periderm tissues: Ck, cork; Pg, phellogen; Pd, phelloderm (cracks occurred during preparation). (**c**) Gall on August 15. Strongly developed phelloderm. Lateral nutritive tissue (Nt2) was scarce (Sc, sclerenchymatous layer); (**d**) (SEM). The appendices (hairs) are the transformed epidermal cells; Cp, central plane of the gall. Arrows on pictures indicate the correct region so that it is not covered by the marking.

A more developed gall of *N. numismalis* showed multiple calcium oxalate crystals (CaOx) accompanying the layer of sclerenchyma surrounding the larval chamber (Figure 7). The chamber had a form of lenticular capsule on its cross-section. The band of the CaOx crystals became thicker during gall development.

The cross-section shown in Figure 7b shows the gall in which the tissues of the periderm (phellogen, cork, and phelloderm) are very distinct. The gall shown in Figure 7c was collected on August 15th. The phellogen was barely visible, and its activity was possibly terminated. The stratum of the cork was distinct and strongly stained with Sudan III. The stratum of the phelloderm was very thick. The nutritive tissue situated above and below the larva was almost completely consumed; new nutritive tissue started to form at the corners of the lenticular sclerenchyma capsule (Nt2) (Figure 7c). Figure 7d shows the border of the central part of N, numismalis gall; the hairs covering the sides of the gall are transformed cells of the epidermis.

**Figure 8:**
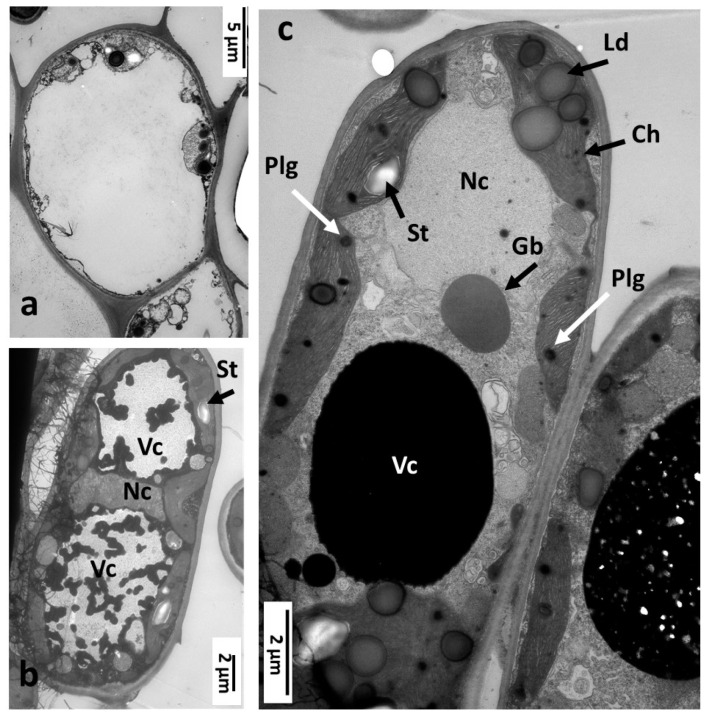
Neuroterus. numismalis gall (TEM). (**a**,**b**) Examples of phelloderm cells. Nc, nucleus; Vc, vacuole; St, starch. (**c**) Fragment of another phelloderm cell, Ld, droplets of lipids; Ch, transformed chloroplast without chlorophyl; Plg, plastoglobules; Gb, “gray body” of unknown function.

Phelloderm is the dominant tissue in *N. numismalis* gall. The cells were relatively large (10–25 µm), round or oval, and had large vacuoles occupying almost the entire interior of the cell (Figure 8a,b). A thin layer of cytoplasm was lining the cell walls. In the cell (Figure 8b), there was a nucleus and large vacuoles with dense osmophilic substances (tannins), and amyloplasts with starch grains were contiguous to the cell walls.

#### 3.3.3. The Nutritive Tissue of *Neuroterus numismalis*

**Figure 9:**
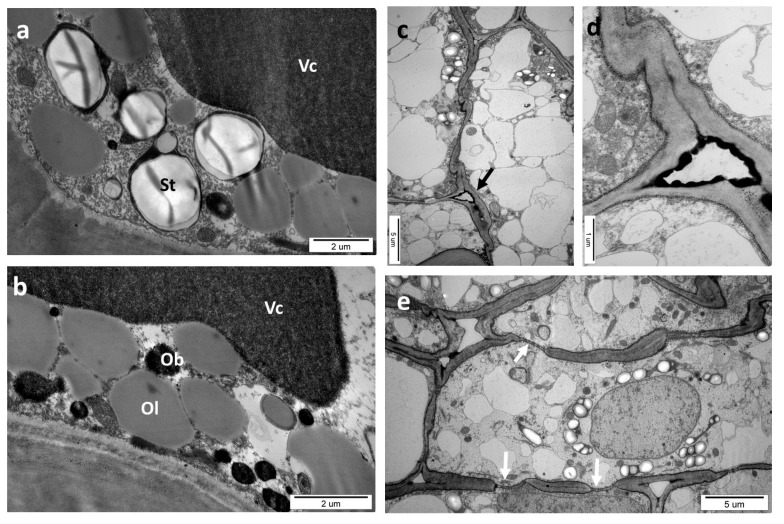
Nutritive tissue of *Neuroterus numismalis* gall (TEM). (**a**) Fragment of the cell with starch grains in amyloplasts (St). (**b**) Cell with numerous droplets of oil (Ol) and of osmophilic bodies (Ob) dispersed in the cytoplasm; osmophilic material also filled the vacuoles (Vc). (**c**–**e**) Other phelloderm cells. (**c**) Visible fragmented vacuoles. Black arow—intercellular space contains osmophilic substance. (**d**) Osmophilic substance higher magnification. (**e**) Other phelloderm cell: garland of amyloplasts surrounding the nucleus. White arrows: primary pit fields.

The cells of nutritive tissue in the cross-section contained multiple round, dark gray bodies with a diameter of approximately 1–4 µm showing a homogenous internal structure (Figure 9a,b). They were not covered by any visible membranes; supposedly, they were oil droplets. There were also numerous small osmophilic bodies (about 0.5–1 µm) which we consider to be the aggregations of tannins. Plastids (amyloplasts) containing large starch grains (usually one per amyloplast) were also present (diameter: approximately 1–4 µm) (Figure 9a). A small amount of amyloplast stroma is visible around the starch grains. Large vacuoles (Vc) filled with tannins were also observed in these cells (Figure 9a,b).

### 3.4. The Gall of Cynips (Diplolepis) longiventris Hartig

**Figure 10:**
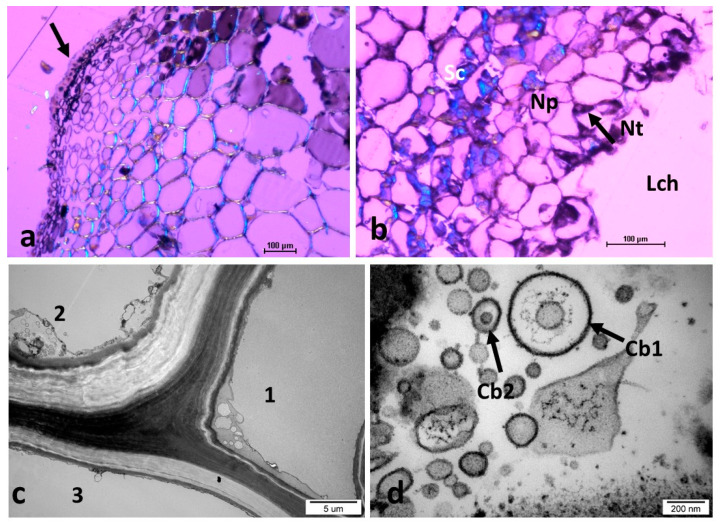
Cynips longiventris gall ((**a**,**b**): LM, polarized light; (**c**,**d**): TEM). (**a**) Gall parenchyma has large cells. They become smaller near the comb (arrow). (**b**) Cells near the larval chamber (Lch): Nt, nutritive tissue; Np, nutritive parenchyma; Sc, sclerenchyma. (**c**) Fragment of the gall parenchyma: thin-walled cell (1) contiguous to two thick-walled cells (2, 3). There were three distinct layers in the walls. (**d**) A fragment of the cytoplasm from cell 2 with various circular bodies. Among them were Cb1 and Cb2.

The galls of this species are relatively large (approximately 8–12 mm) (Figure 1c). The cells of the main body were up to 200 µm in diameter (Figure 10a). They were highly vacuolated, with a thin layer of cytoplasm lining their walls. There was one or more colorful combs on the surface of the gall. The cells situated near the comb are smaller (Figure 10a, black arrow). The larval chamber was surrounded by a stratum of nutritive tissue containing dense cytoplasm (Figure 10b). The next, more external strata were “nutritive parenchyma”, sclerenchyma, and gall parenchyma.

The gall of *C. longiventris* is hard due to its cell wall structure. They were about 1.7–8 µm thick and composed of three layers (Figure 10c). The layer contiguous to the cytoplasm was little transparent to the electrons; the next layer was transparent to the electrons and in cell “2” it is thick, which would mean that this cell belongs to sclerenchyma. The most internal layer of the cell wall was moderately transparent to electrons and was the primary cell wall.

The cytoplasmic layer lining the walls showed various bodies in TEM (Figure 10d). For instance, there was a round body (Cb1) with a diameter of approximately 400 nm, and the central core was also round (with a diameter of approximately 160 nm) with a slightly darker (less translucent for electrons) content. The body and its central round core were surrounded by double membranes. The outer double membrane exhibited rough, dark material on its internal and external sides. Right next to this body, there was another similar (Cb2) one, but this was smaller. In addition, there were several other round bodies with a diameter of approximately 50–200 nm which had various electronic densities. They were covered with either single or double membranes (Figure 10d). The bodies shown in Figure 10d, surrounded by single or double membranes, were similar to the formations related to the autophagic and endocytic pathways in plants.

**Figure 11:**
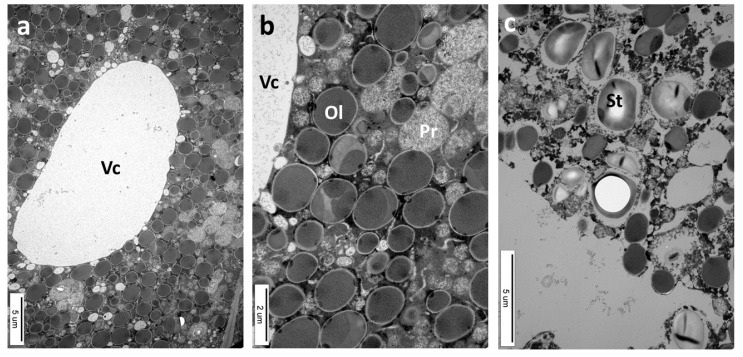
Cynips longiventris gall (TEM). (**a**–**c**) The nutritive tissue loaded with nutrients: Vc, vacuole. (**b**) Fragment of (**a**) at higher magnification: Ol, probably droplets of oil; Pr, probably proteinaceous bodies—there are two sizes: large and small. (**c**) St, starch grains.

The cells of the nutritive tissue of *C. longiventris* were rich in nutrients (Figure 11a–c, TEM). Round dark gray bodies (1–2 µm), with a homogenous content, each surrounded by a thin, clear areola, were oil droplets (Ol). Light-gray bodies with a granular structure were also observed. They were of two different sizes: the larger ones were approximately 2.2 µm and the smaller ones 0.4–0.6 µm. They were probably proteinaceous in nature. Some cells also contained amyloplasts with starch grains (diameter approximately 2.5 µm). The small black round bodies (approximately 2 µm) in Figure 11c were probably granules of tannins.

**Figure 12:**
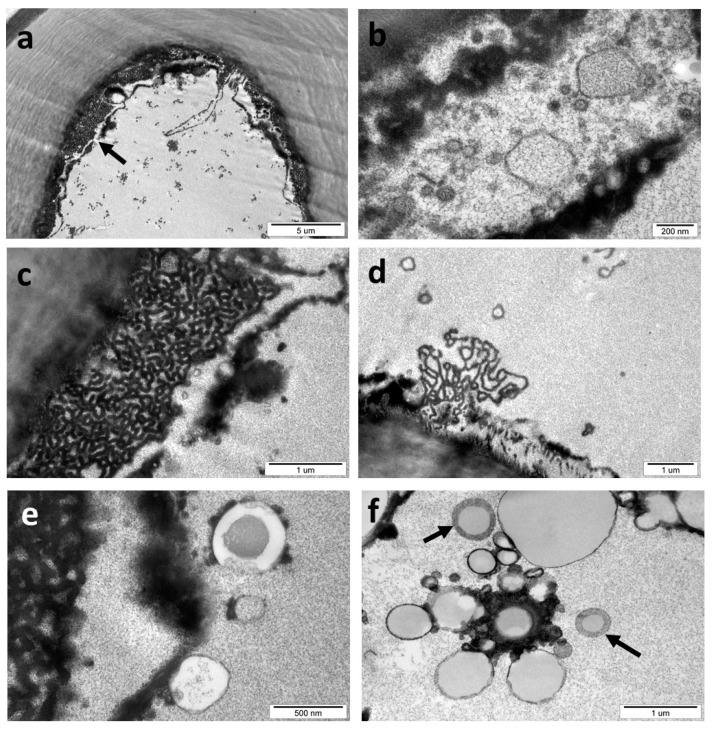
Cynips longiventris gall (TEM). (**a**) Cell of the gall sclerenchyma—the fragment of cytoplasm indicated with an arrow is shown separately in figures (**c**–**f**). (**b**) Microbodies. (**c,d**) The labyrinth-like body of the interwoven membranes is “an organized smooth endoplasmic reticulum”. (**e**) Autophagosomes. (**f**) The group of vesicles joined by electron-dense substances—we do not know their role or the role of the two round bodies marked by arrows.

In still-live sclerenchyma cells of *C. longiventris* gall, different bodies were found in the cytoplasm (Figure 12). Two bodies of an irregular shape surrounded by a single membrane (Figure 12b) are probably microbodies; however, their true nature can only be verified by chemical methods [38]. The labyrinth-like fragments in Figure 12c,d are very similar to the “organized smooth endoplasmic reticulum”, described by Ferrero et al. [39]. They probably have a glandular function. In some sclerenchyma cells (Figure 12e), round (probably spherical) bodies (diameter 260–500 nm) with a slightly osmophilic core were surrounded by a wide electron-clear envelope. The nature and role of electron-dense bodies with irregular shapes and several vesicles of different sizes on their periphery (Figure 12f) are unknown. In the same image, there are two round bodies (see the arrows) with an electron-clear center and an electron-dense envelope (diameter of approximately 350–600 nm).

**Figure 13:**
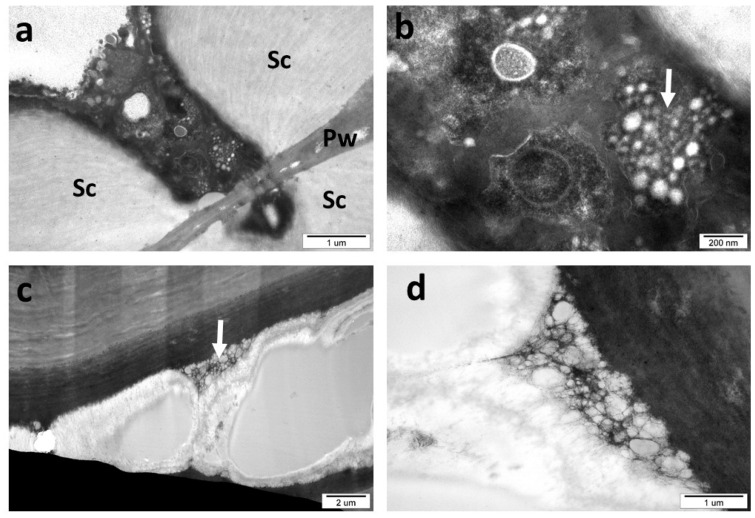
*C. longiventris* gall (TEM). (**a**) A strip of cytoplasm between very thick sclerenchyma walls (Sc), Pw, primary wall. (**b**) A detail of the previous picture. The white arrow shows a group of very small vesicles in higher magnification. (**c**) Swallen middle lamella in gall. An arrow—its granular structure. (**d**) Middle lamella in higher magnification.

Sclerenchyma cells sometimes contained in their cytoplasm a large group of very small (15–80 nm) vesicles (Figure 13a,b). This group was not surrounded by any visible membrane. Their role is unknown. The middle lamella in the *C. longiventris* gall is normally almost invisible, but in some cases, it is markedly swollen and shows a granular structure.

## 4. Discussion

In the present study, we described the anatomical and ultrastructural properties of the galls of three monophagous Cynipidae wasps that feed on pedunculate oak leaves. However, the galls of each species exhibit distinct anatomical and ultrastructural characteristics.

The structure and development of the *N. numismalis* gall during the early stages of its development are similar to that described by Hough [40] and Meyer and Maresquelle [4] for a very similar species called *N. quercusbaccarum*. The egg is laid into the leaf tissues and the larva hatches and moves into the plasteme (new, not yet differentiated tissue formed in response to the galling insect). Some plasteme cells are subjected to lysis, forming a place for the larval chamber [16,40]. The larva enters it. The larvae of several other Cynipidae behave similarly [4].

We examined the point of coalescence at the abutment between two different types of tissues: tissues of the leaf blade of the host and those of the gall. As an example, we chose the coalescence point of *C. quercusfolii* gall because it is easily accessible and relatively large. An important aspect of the coalescence point is that the different vascular bundles may have different roles. Some of them probably only pass through the point of coalescence and do not participate in providing food for the gall. They are mainly destined to supply water and nutrients to the distal parts of the leaf behind gall insertion. At the center of the point of coalescence, the vascular strands of a gall and those of a leaf are situated in close proximity, facilitating the potential exchange of nutrients and water between them. Meyer and Maresquelle [4] mentioned the coalescence point in gall species that abscise; however, the gall of *C. quercusfolii* does not abscise from the leaf but is shed with a whole leaf.

The original attribute of the *C. quercusfolii* gall is the construction of the larval chamber in the final stage of larva development. It was formed of the rest of nutritive cells consumed earlier by the larva. A similar case was described by Rohfritsch [10].

The was unique was the gall parenchyma in *C. quercusfolii* composed of giant thin-walled cells with huge vacuoles and a very small nucleus and nucleolus. Cell wall thickening is occasionally observed at the junctions between three or four cells. This structural feature likely contributed to tissue strengthening. In certain instances, sections of the cell walls were significantly swollen and exhibited a highly complex structure (Figure 4h). The function of these structures remains unclear. One hypothesis suggests a potential glandular role, while a phytopathological cause cannot be ruled out.

Characteristic localization of the meristematic region was observed in a young *N. numismalis* gall. Meristematic cells containing a dense cytoplasm were situated around the gall stalk, and subsequently, the meristematic activity gradually shifted toward the margin of the gall disk. This localization of the meristematic region is associated with the mechanism of gall growth. The micrographs obtained under fluorescent light indicated that the cells of the leaf situated around the gall stalk exhibited pronounced lignification (Figure 6b,c). This phenomenon may enhance the structural integrity of the gall’s attachment to the leaf blade.

The phelloderm cells in *N. numismalis* galls frequently exhibit fragmented vacuoles [41]. A similar phenomenon was observed by LeBlanc and Lacroix [42] as an early effect of the oviposition fluid of the galling insect (*Diplolepis rosefolii*). Vacuole fragmentation is considered a normal occurrence in trees preparing for winter dormancy, and it reverses in spring [43].

In a typical oak leaf, vacuoles frequently contain electron-opaque precipitates. According to Brillouet et al. [44], these precipitates are tannins. Tannins were present in both healthy oak leaf tissues and galls. They manifested in punctate or granular form or as large globules with a uniform internal structure filling the vacuoles. Such globules occasionally exhibited small “holes” (resulting from uneven disposition of pigment) on a cross-section. Tannins serve numerous functions in plants [45]. When they accumulate in the gall external tissues (epidermis and the nearest layers of the parenchyma), they play a protective role, defending the larva against invading organisms or inquilines owing to their toxic or deterrent properties.

The results presented by us showed a striking difference between the gall parenchyma and nutritive tissue (Figure 5). Such differences were also observed by many previous authors [4]. The cells of the nutritive tissue of *C. quercusfolii* exhibited dense cytoplasm, large nuclei, and prominent nucleoli, suggesting an enhanced capacity for protein synthesis. The role of nutritive tissue is therefore not only to temporarily store nutrients and cell building materials but to also produce different proteins or other nutritive or building materials. In cross-sections of nutritive tissue of the galls *N. numismalis* and *C. longiventris,* many oil droplets, starch grains, proteinaceous bodies, and tannin granules were observed. This indicates that the larvae have an abundant source of food in the nutritive tissue.

The gall is a strong sink for nutrients. They are attracted from the whole leaf blade to which the gall is attached [46] and sometimes from more remote parts of a plant [47,48]. Our results with *N. numismalis* and. *C. longiventris* galls show that nutritive tissue is a rich source of food for a larva. We found that the type of storage material in nutritive tissue could differ between the galls of each wasp species and between the galls of the same species at different stages of development. Proteins were present in the nutritive tissue of the *C. quercusfolii* gall. The accumulation of starch in the nutritional tissues of galls produced by Cynipidae has been reported (e.g., numerous amyloplasts in *C. quercusfolii*) [49]; however, a lack of starch in the nutritive tissue was also observed [41]. In mature *C. longiventris* galls, the cells of the nutritive tissue are filled with lipid bodies. This suggests that the storage material, which is the base of food for the larva, is related to its nutritional requirements and could be adapted to a stage of larval development. The following problem remains: is the mechanism of nutrient attraction to galls similar to that which causes the accumulation of nutrients in fruits or seeds?

The structures similar to autophagosomes [50,51] were present in gall parenchyma cells. These organelles are related to the autophagic pathway in plants. Bozhkov [52] provided evidence that autophagy similar to that in animals is required for the deposition of lipid droplets in plants.

“Labyrinth-like” bodies of a membranous character similar to those described by Ferrero et al. [39] occurred in the sclerenchyma cells of *C. longiventris*, which have not fully terminated the process of sclerification. These structures were named “organized smooth endoplasmic reticulum” (OSER) [53]. They are destined for specific biosynthetic and secretory functions. The role of the OSER structures remains unclear. Sandor et al. [53] says that we do not know what the physiological functions of the OSER are conclusively. This may play an important role in the process of compartmentalization in cells.

The protection of larvae is an important function of plant galls. Robust protective structures were observed in the galls of *N. numismalis*. The protective barriers comprised not only lignified trichomes or sclerenchymatous capsules surrounding the larval chamber but also periderm tissues, such as cork, phellogen, and phelloderm, which collectively reduced the likelihood of invasion or physical injury.

Periderm tissues constitute a large part of the *N. numismalis* gall. Normally, these tissues are present in the stem, and only in exceptional cases are they found in leaves [54]. Meyer and Maresquelle [4] reported that in *N. numismalis*, cork occurred on the outer (lower) gall surface. They do not mention other periderm tissues. The presence of periderm was shown by Kraus et al. [55] in a hymenopteran gall encountered in *Struthantus vulgaris* (Loranthaceae). Meyer and Maresquelle [4] also stated that the formation of the periderm may appear as a reaction to wounding, but in our case, it was closely connected with normal gall development.

In addition to the periderm and sclerenchyma capsule, the protective barrier around the larval chamber was strengthened by the CaOx crystals. Although Paiva [56] claimed that the main role of CaOx is the excretion of solid wastes, such as Ca salts, in the case of plant galls, their additional role may be to strengthen the protection of the larval chamber against the invasion of predators or inquilines. Francheschi and Nakata [57] also name several other possible roles of CaOx, such as the regulation of Ca^2+^ levels in cells.

We have on occasion found in TEM preparations bodies that we could not identify. For instance, in partly sclerified cells of *C. longiventris*, a group of very small vesicles are situated loosely in the cytoplasm and not surrounded by any membrane.

Another interesting case was an extraordinarily swollen middle lamella. Normally, the middle lamella is very thin and difficult to perceive. Some swelling of the middle lamella was observed in the abscission zone of leaves, which prepared themselves to be shed [58].

This study aimed to present a novel observation that complements the existing description of the gall structure and ultrastructure. Three common species of galling insects were chosen as models for our investigation. The insects imposed species-specific morphogenetic programs on the oak tissues. This program involved numerous regulatory elements (derived from insects). Consequently, diverse morphologies arise from the same host tissue. Recently, the main effort in the gall investigation has focused on the application of molecular methods. However, the molecular point of view needs to be supplemented by the results of anatomical and ultrastructural investigations in order to know what real changes the insect introduces to a host. We hope that our study will contribute to this field of research.

## Data Availability

The data generated during this study are reported in the manuscript.
